# Acquired factor XIII deficiency: A scoping review

**DOI:** 10.1097/EA9.0000000000000035

**Published:** 2023-09-29

**Authors:** Olivier Duranteau, Guney Tatar, Anne Demulder, Turgay Tuna

**Affiliations:** From Anesthesiology Service Department, CUB-ULB Hôpital Erasme, Brussels, Belgium (OD, GT, TT), Intensive Care Unit, Percy Military Training Hospital, Clamart, France (OD), Faculté de Médecine, Université Libre de Bruxelles, Brussels, Belgium (OD, AD, TT), Laboratory of Hematology LHUB-ULB ULB, Université Libre de Bruxelles, Brussels, Belgium (AD)

## Abstract

Acquired factor XIII (FXIII) deficiency is a very rare haemostatic defect that can be either immune (rare development of an autoantibody targeting FXIII epitopes) or nonimmune (diminished synthesis or increased consumption of the same factor). The aim of this study is to review the symptomatology, the diagnostic method used, but above all to determine the most frequently used and potentially most effective treatment for acquired FXIII deficiency.

PubMed, Medline, embase/Ovid databases were queried from 1 January 2012 to 3 April 2022. Data extraction was performed using the keywords ‘Acquired FXIII deficiency.’

The systematic search identified 474 records. After screening titles and abstracts, only 36 articles met the eligibility criteria.

The mean age of all patients was 57.6 [range, 1–84] years. The male to female ratio was 35 : 25.

The majority of cases described were due an autoimmune reaction with antibody production (24 manuscripts), only six manuscripts described consumption. The most prevalent symptoms were local haematoma (31).

Six cases died, two from haemorrhagic shock, two from haemorrhagic stroke, one from respiratory distress, and 1 from septic shock.

Given the patient outcomes, this review confirms that the most appropriate treatment consists of one of the following elements or a combination of several of these elements: FXIII concentrate, corticosteroids, cyclophosphamide, rituximab for autoimmune cases, and FXIII concentrate supplementation only in case of consumption.

**KEY POINTS**

- Only 36 articles from 2012 to 2022 describe factor XIII (FXIII) acquired deficiency and its management.
- Of these 36 manuscripts, 24 described cases due to antibody production, 9 described consumption, and 3 manuscripts did not described the cause of the deficiency.
- The most prevalent symptoms were local haematoma (31) followed by uncontrolled general bleeding (8).
- In cases with FXIII deficiency due to autoimmunity, the main treatment consisted of FXIII concentrate, corticosteroids, cyclophosphamide and/or rituximab alone or combined.
- In cases with FXIII deficiency due to consumption, supplementation was performed with FXIII concentrate only.

## Introduction

**FIGURE 1:**
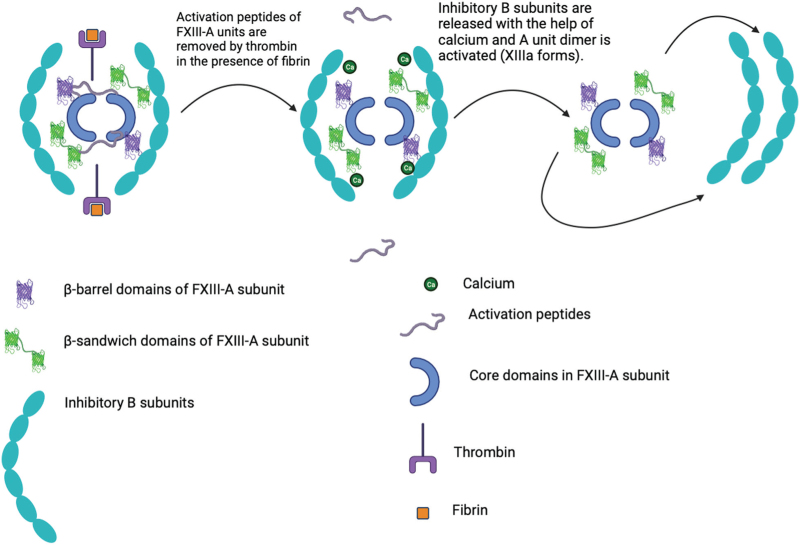
Factor XIII activation process by thrombin in presence of fibrin and calcium.

Haemostasis comprises a set of physiological mechanisms that prevent or stop bleeding while maintaining the fluidity of circulating blood. Among these mechanisms, plasma coagulation allows consolidation of the platelet plug, the first barrier to bleeding, by the final formation of thrombin. Thrombin ends the cascade of reactions by converting fibrinogen to fibrin and by activating factor XIII (FXIII).^1^ This essential factor comprises two subunits, A and B; the A subunit contains the catalytic activity while the B subunit mainly acts as a transporter/carrier in plasma.^2^ Activated FXIII cross-links alpha and gamma fibrin chains allowing the critical role of clot stability, but also induces resistance to fibrinolysis and protects against proteolysis in the last step of the coagulation cascade^3^ (Fig. 1). Given the essential role of this factor in haemostasis, a deficiency in FXIII has considerable clinical consequences. The diagnosis cannot be suspected on the basis of prothrombin time (PT) or activated partial thromboplastin time (aPTT) as these measurements remain normal regardless of the severity of the deficit. Diagnosis therefore remains based on the specific dosage of the factor (using a specific reagent) and on the symptomatology (uncontrolled bleeding). The measurement of FXIII activity is based on the use of a reagent specific for FXIII, whereby the reaction between the blood of an affected patient is compared with a control blood sample. A reduction in activity to less than 70% of normal is considered abnormal.^3^ Morbidity is increased by the acquired FXIII deficiency, including prolonged intensive care stays and higher incidence of major bleeding.^4^ The biological cut-off point for defining a disability is usually 70%.^3^ Morbidity is increased by the acquired FXIII deficiency, including prolonged intensive care stays and a greater incidence of major bleeding.^4^

The congenital form of FXIII deficiency is an extremely rare autosomal recessive disorder, primarily located in remote mountain regions of Afghanistan and Iran, and is due to consanguinity. Clinical features of the deficiency are typical and characterised by umbilical stump bleeding, spontaneous/delayed bleeding, intracranial haemorrhage, unusual wound healing, and a defect in scar formation.

Acquired deficiency is due to two aetiologies: immune causes due to the rare development of autoantibodies targeting FXIII epitopes, and nonimmune causes due to accelerated consumption.^5,6^ Given the extreme rarity of the acquired deficiency, it is usually underdiagnosed. Conventional clinical studies are not feasible, so the prevalence of the condition is unknown.^5^ Nevertheless, an increasing number of case reports, case series, and articles are being published.

This review article focuses on the management and treatment strategies for acquired FXIII deficiency in the medical and perioperative settings.

## Materials and methods

### Data sources

The completeness of case report collection was evaluated using CAse REport 2013 (CARE) guidelines as a template.^7^

This scoping review was conducted consulting the databases Medline, embase/Ovid, and PubMed, covering a period ranging from 1 January 2012 to 3 April 2022 with ‘Acquired FXIII deficiency’ as the keyword. Articles other than in French or English were translated with DeepL Translator (DeepL SE, Cologne, Germany).

The initial search was based on the study published in 2016 by Tone *et al.*^8^ The period of inclusion is later than that of Tone, and does not include the articles included that publication. In the study,^8^ articles were referred back to 2014. However, we found seven other publications on the topic between 2012 and 2014 that were not yet included in the above-mentioned study.^8^ We therefore chose to take the starting year as 2012.

### Eligibility criteria

We included in our analysis peer-reviewed articles, case reports, case series, and letters (citing acquired deficiency proven by factor dosage). A FXIII activity level of 70% was used to define a deficiency. However, some publications used higher thresholds and these were included because the patients were treated as FXIII deficient. Articles on congenital FXIII deficiency, knock-out mice with FXIII deficiency, new diagnostic methods for deficiencies, molecular biochemistry of FXIII, as well as nonhuman articles and abstract only were excluded.

### Study selection

Two independent reviewers (O.D., G.T.) used systematic review data repository (SRDR+) software from the Agency for Healthcare Research and Quality (AHRQ) and screened titles, abstracts, and full texts queried by the systematic search. These two authors, blinded to each other's results, also performed the search according to the above recommendations. Selection was based solely on the title and abstract of a study. If a discrepancy between the two searches appeared, a third more experienced author (T.T.), made the final selection. During the collection process, the recommendations of the Joanna Briggs Institute were followed.^9^

### Data abstraction and synthesis

Two independent reviewers (O.D., G.T.) manually extracted the data from each full text using a standardised Excel spreadsheet. This spreadsheet included patient age and sex, clinical symptoms, past bleeding history, relevant medical history (e.g. associated autoimmune, cardiac or oncological pathologies), medical or preoperative settings, interventions, outcome posttreatment, baseline laboratory values (haemoglobin, platelets, INR, aPTT, fibrinogen), FXIII activity (baseline and postintervention), inhibitor type and levels (baseline and postintervention).

### Assessment of completeness of reporting

Completeness of case report collection was evaluated using CAse REport 2013 (CARE) guidelines as template^7^ and assessed using the 13-item checklist recommended by the ‘Enhancing Quality and Transparency of Health Research Network’ (EQUATOR). Each item was subdivided into 26 individual checklists. Items have been reported in exactly the same way as they were described in the original article. One item (prognosis) was removed because no significant predictive outcomes of acquired FXIII deficiency exist. Final eligible case reports were reviewed by two independent reviewers (O.D., G.T.), and discrepancies were addressed through discussion with the third reviewer (T.T.). Reporting of the results was then expressed as percentages.

## Results

### Study selection

**FIGURE 2:**
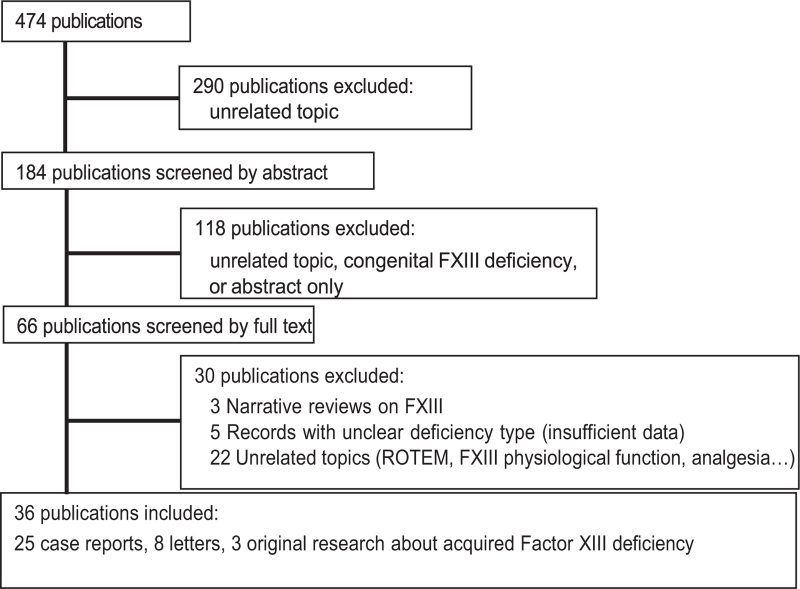
Systematic reviews article selection flow diagram.

The systematic search identified 474 records. After screening titles and abstracts, only 36 articles met the preset eligibility criteria and underwent the reviewers’ complete analysis and data extraction (Fig. 2). No duplicate articles were found.

### Patient characteristics

From the 36 publications selected, 60 patients were found in 24 case reports,^5,10–32^ four manuscripts referenced as articles,^2,33–35^ and eight letters to the editor.^36–43^

There were 48 patients in a medical setting^2,10,11,20–30,33–37,40–43^ and 13 in a surgical (perioperative) setting.^5,12–19,31,32,38,39^ One trauma patient had been included in the surgical patient category,^12^ and there were no obstetric case described. Most of the patients were from Asia, although it should be noted that Lichter's study^33^ alone reported a total of 29 patients from Israel, all SARS-COV2 positive. The mean age of the patients was 56.6 [range, 1–84] years. The male to female ratio was 35 : 25. Patient characteristics are summarised in Table 1.

**Table 1:** Patient characteristics

			Medical (*n* = 24)	Surgical (*n* = 12)	Combined (*n* = 36)
Studies	Case reports		15	10	25
	Journals		3	0	3
	Letters		6	2	8
		Participants	48	12	60
		Age (mean)	57.6	55.8	56.7
		Age [range]	[1 to 84]	[10 to 82]	[1 to 84]
		Sex M/F	29/19	6/6	35/25
Reported symptoms	Haematoma	Muscular	15	5	20
		Subcutaneous	5	3	11
		Cranial	2	2	4
		Retroperitoneal	2	0	3
		Peri-oral	0	1	1
		Splenic	1	0	1
	Haemorrhage	Digestive	6	1	7
		Cerebral	3	3	6
		Subcutaneous	1	2	3
		Tonsillar	0	1	1
		Sublingual	0	1	1
		Vaginal	1	0	1
		Intraarticular	1	0	1
	Purpura		3	2	5
	Ecchymosis		3	0	3
	DVT		2	0	2
	Petechiae		1	1	2
	Respiratory distress		1	0	1
	Pseudomeningocoele		0	1	1
	Haemoptysis		1	0	1
	Pulmonary Embolism		1	0	1
	Cardiac Tamponade		1	0	1
	Haematuria		0	1	1
Risk factors	Endocrine		3	3	6
	AutoImmune		4	1	5
	Cardiac		4	1	5
	Cancer		2	3	5
	Surgery		2	2	4
	Renal		3	1	4
	Medication		2	2	4
	Liver disease		1	2	3
	Digestive		1	1	2
	SLE		2	2	2
	None		8	1	9
	Not reported		2	1	3
Reported bleeding history	Undefined bleeding		2	2	4
	Dental		2	0	2
	Menorrhagia		1	1	2
	Cerebral		0	1	1
	Surgical		1	0	1
	Parturition		1	0	1
	No bleeding history		14	9	23
	Not reported		3	1	4

DVT, deep vein thrombosis; SLE, systemic lupus erythematosus.

### Clinical presentation

All patients were symptomatic. The most prevalent reported symptom was haematoma, including 20 muscular,^2,15,17–20,24–29,32,35,36,40–43^ and 11 subcutaneous^5,12,19,25,30,34,37–40,42^ (Table 1). Concerning bleeding during surgery cases, if it is related to the surgery, it is indicated as surgical in Table 2, if it is not related to the surgery it is indicated as medical.

**Table 2:** Laboratory data: baseline and postoperative results (in parenthesis) were separated if necessary

		Medical (*n* = 23)	Surgical (*n* = 13)	Combined (*n* = 36)
Reported results	Haemoglobin (abnormal)	18 (17)	9 (7)	27 (24)
	Platelets	10 (1)	8 (1)	18 (2)
	INR	5 (1)	4 (1)	9 (2)
	aPTT	12 (2)	6	18 (2)
	Fibrinogen	8 (2)	7 (5)	15 (7)
Baseline results	FXIII activity	24	12	36
	Inhibitor measured	19	5	24
Postintervention results	FXIII activity	19	10	29

aPTT, activated partial thromboplastin clotting time; INR, prothrombin time, international normalised ratio.

Twenty-seven cases reported initial haemoglobin values, with 24 of them being abnormal. Coagulation results were sparsely reported: platelet count (18 reported, 2 abnormal), INR (9 reported, 2 abnormal), aPTT (18 reported, 2 abnormal), and fibrinogen (15 reported, 7 abnormal). Exact numbers are presented in Table 2: in individual cases where the FXIII level was simply stated to be ‘normal’, we did not included these in calculations.

### Risk factors and intercurrent Illness

Twenty-three articles reported the presence of a risk factor. Endocrine disturbances (thyroid and obesity) were the reported risk factor in six reports,^22,29,32,36,38,39^ followed by five autoimmune diseases (rheumatoid arthritis,^19,35^ psoriasis,^21^ primitive amyloidosis,^40^ Henoch-Schoenlein vasculitis^43^), five cardiovascular conditions (heart disease,^24,36^ aortic dissection,^12^ atrial fibrillation,^22,24^ and hypertension,^24,27,36^ and five cancers (leukaemia,^12,37,39^ adenocarcinoma,^10^ and lymphoma^27^). We also found surgery (total hip replacement),^10,12,19,42^ renal conditions (kidney disease and nephrotic syndrome,^13,22,25,43^ and drugs (tocilizumab,^19,35^ vanoprazan,^31^ and domperidone^2^) as risk factors associated with FXIII deficiency. Digestive (ulcers^31^ and colitis^43^) and systemic lupus erythematosus^25,43^ were the least frequent risk factors. Four cases did not report any risk factor,^23,26,28,30^ and nine reported an absence of any risk factors.^5,10,11,15,16,20,34,38,42^

### Previous bleeding histories

Only the descriptions of four patients did not mention whether or not they had a history of bleeding.^13,21,22,24^ Two out of the 12 patients in the perioperative setting presented with easy bleeding/bruising, one with cerebral bleeding^38^ and one had bleeding with menorrhagia.^39^ Twenty-one medical patients reported a bleeding history, two patients with undescribed bleeding,^27,36^ one patient with previous surgical bleeding,^40^ two patients with bleeding during dental extraction^30,34^ and one patient with menorrhagia.^27^ Fourteen medical^2,5,10,11,20,23,26,29,33,35,37,41–43^ and nine per-operative patients had no previous history of bleeding.^12,13,15,16,18,19,32,33^

### Laboratory diagnosis

Acquired FXIII deficiency was established in 59 patients by measuring the FXIII activity using chromogenic or nitrogen release assay. One patient was diagnosed using FXIII-A antigen assay.^39^ Six publications failed to report any FXIII activity level posttreatment.^19,22,27,33,35,39^ As for post treatment FXIII activity, only 29 mentioned the level.^5,10–12,14–16,18–20,22–24,26–28,30,33,36–42^ Baseline FXIII activity ranged from 0 to 79%, while posttreatment FXIII activity ranged from 1 to 249%.

Twenty-four cases reported the presence of an inhibitor, nine cases reported absence of inhibitor^12,13,15,16,31,33,35,37,39^ (these patients were classified as having FXIII deficiency by exclusion (absence of inhibitor) after having tested platelet function and various coagulation factors), three publications did not report any measurement of FXIII inhibitor titre^14,21,38^ (Tables 2 and 3).

**Table 3:** Interventions and outcomes

	Aetiology			Case		Treatment														Outcome	
Ref	Author	Antibodies	Consumption	Unknown	Surgical	Medical	FXIII concentrates	Prednisolone	Cyclophosphamide	Rituximab	Plasmapherisis	Immunoglobulins	Bortezomib	FFP	RBC	DDAVP	F VIIa	Cryoprecipitate	No treatments	Resolution / stop the bleeding	Death
2	Amano S	x				x		x												x	
5	Hayashi T	x			x		x	x												x	
10	Kun M	x				x	x	x	x	x	x									x	
11	Bovet J	x				x		x	x	x		x	x							x	
12	Wakabayashi N		x		x		x														x
13	Yamada Y		x		x		x													x	
14	YAMANISHI S			x	x														x		x
15	Takashima Y		x		x		x													x	
16	Jankovic M		x		x		x							x	x	x				x	
17	Tone K	x			x		x	x		x	x			x						x	
18	Kojima M	x			x		x	x	x											x	
19	Matsuoka M	x			x		x	x	x											x	
20	Shimoyama S	x				x	x	x				x								x	
21	d’Angelo DM			x		x						x								x	
22	Di Micco P	x				x								x			x			x	
23	Murata M	x				x	x													x	
24	Soto I	x				x	x	x		x										x	
25	Rabik CA	x				x	x	x	x		x	x		x						x	
26	Pénzes K	x				x		x	x	x				x			x	x		x	
27	Nixon CP	x				x		x		x								x			x
28	Kotake T	x				x	x		x	x										x	
29	Boehlen F	x				x	x										x			NA	
30	Mitchell JL	x				x	x	x												x	
31	Takamatsu N		x		x		x													x	
32	Uchida E	x			x			x	x												x
33	Lichter Y		x			x													x	x	
34	Kessel R	x				x		x									x			x	
35	Mokuda S		x			x	x													x	
36	Beckman JD	x				x	x	x													x
37	Feugray G		x			x	x														x
38	Tsuda M			x	x			x										x		x	
39	Gonçalves E		x		x		x													x	
40	Ferretti A	x				x	x							x						x	
41	Tang N	x				x		x	x									x		x	
42	Ngo Sack F	x				x				x							x			x	
43	Tha MH	x				x		x				x		x			x	x		x	
	Total	24	9	3	13	23	22	19	9	8	3	5	1	7	1	1	6	5		29	6

DDAVP, desmopressin acetate; FFP, fresh frozen plasma; RBC, packed red blood cells.

Baseline inhibitor titres ranged from 0 to 100 Bethesda units ml^−1^ (BU ml^−1^), while posttreatment inhibitor ranged from 0 to 40 BU ml^−1^: 1 BU ml^−1^ is required to inhibit 50% of FXIII activity.

### Therapy

The majority of patients whose acquired deficiency was due to high consumption were treated with FXIII concentrate only,^12,13,15,23,35,31,37,39^ and only one reported case received a combined transfusion of fresh frozen plasma, red blood cell, and platelet concentrate.^16^ All treatments are summarised in Table 3.

Regarding auto-immune cases, many different treatments, combination of treatments, or no treatment at all were described (Table 3). Twenty-four manuscripts described patients receiving combined treatments.^5,10,11,16–20,22,24–30,32,34,36,38,40–43^ In view of the different combinations observed, the treatment baseline appears to be based on a combination of FXIII concentrate and corticosteroids. Cyclophosphamide and rituximab were the most frequent additions.

### Outcomes

Of the 36 publications, 35 reported outcomes (Table 3): six reported death,^12,14,27,32,36,37^ 29 reported full cessation of bleeding following FXIII activity increase after treatment and were discharged.^2,5,10,11,13,15–26,28,30,31,33,34,38,35,39–43^ One manuscript reported cases of patients with COVID 19 and diagnosed FXIII deficiency.^29^

## Discussion

This scoping review presents an extensive dataset spanning from 2012 to 2022, focusing on patients with confirmed acquired FXIII deficiency in medical and perioperative settings. FXIII deficiency is a rare condition that is associated with significant morbidity and mortality. The clinical presentation of this condition is perplexing, as it involves the occurrence of haematomas despite normal coagulation and platelet function. Through this scoping review, it becomes evident that acquired FXIII deficiency is characterised by an absence of a significant bleeding history in patients, in contrast to congenital deficiencies where bleeding typically occurs in early infancy.^44^ By compiling a comprehensive database of case reports encompassing patients’ blood tests, symptoms, and treatment strategies, there is potential to enhance the identification and management of this disorder.

Given the limited number of case reports published during the 10-year period covered by this review, it is plausible to assume that this deficiency is largely underdiagnosed or underreported. On average, and except during the COVID-19 crisis, fewer than 10 cases were reported annually, with the majority of cases documented in Japan. However, it is noteworthy that a recent study, published in December 2022 by Hetz *et al.*, provides new data on trauma patients.^45^ This study^45^ reports FXIII activity, even in cases with no haemorrhagic symptoms, and noted that 12.4% of patients had an asymptomatic deficiency. Moreover, the deficiency worsened during the damage control phase, with FXIII activity decreasing from 85% to 58%.

The laboratory diagnosis of acquired FXIII deficiency poses challenges. FXIII activity testing is only available in specialised laboratories. The Japanese Collaborative Research Group (JCRG) recommends diagnosing FXIII deficiency through the determination of the factor's activity. Once confirmed, further testing is required to identify inhibitors and differentiate between immune and nonimmune deficiencies.^46^ Concomitant states of hyperfibrinolysis were described in 2021.^47^ Levels of D-dimer, fibrin degradation products and the plasmin-plasmin inhibitor complex increased, while levels of the α2-plasmin inhibitor decreased during the bleeding period. This mixture of hyperfibrinolysis and hypocoagulability is not described in the various articles and deserves particular attention for clinical practice.

A significant proportion of the described patients originates from East Asia, particularly Japan. A study by Osaki in 2021 sheds light on the potential explanation for this geographical predisposition.^48^ The study suggests that variations in HLA I and II alleles contribute to increased levels of anti-FXIII antibodies. Conducting prospective studies on HLA in acquired FXIII deficiency could enhance our understanding of the disease's origin and geographical tendencies.

Currently, there are no established guidelines for treating acquired FXIII deficiency due to the rarity of the condition.^49^ Patient management involves a two-step process, with the first step entailing immediate haemostatic treatment in cases of excessive bleeding, followed by the identification of the type of FXIII deficiency. Treatment options involve FXIII replacement in cases of nonimmune deficiency and removal of autoantibodies/inhibitors in cases of immune-related disorders. FXIII concentrates such as Fibrogammin-P or NovoThirteen are available for supplementation, but their effectiveness may be limited in the presence of high inhibitor titres. Case reports highlight the challenges associated with inhibitor eradication, even when employing combinations of immunosuppressive therapies. This review reveals that the simultaneous administration of multiple therapies makes it difficult to evaluate the individual benefits of each treatment. While the presence of an inhibitor diminishes the half-life of FXIII, aggressive immunosuppression or inhibitor filtration through plasmapheresis may probably avoid or reduce the need for FXIII concentrate, leading to better outcomes. Our review notes that when FXIII activity was fully restored there was cessation of bleeding in 80% of the patients; however, no follow-up data beyond discharge were documented.

### Limitations

In this study, we employed the CARE guidelines to assess the quality of the case reports utilised in our data extraction process. These guidelines were developed to aid clinicians in submitting comprehensive and transparent case reports by adhering to a 13-point checklist.^7^ However, we made a modification to one item on the checklist, specifically the prognosis component, as no prognostic information was available for FXIII acquired deficiency. While certain baseline details such as age, sex, and symptoms were reported, there were several missing data points, including information on invasive procedures. Furthermore, there was a notable dearth of patient outcomes reported. One striking observation was the lack of documented informed consent, with only 28% of the case reports clearly stating that consent had been obtained for publication.

## Conclusion

The aim of this study was to improve recognition of the under-diagnosed nature of acquired FXIII deficiency. This review highlights the scarcity of data, the difficulties in diagnosis of FXIII deficiency, and the lack of consensus on the treatment.

Depending on the exact cause of the deficiency, the treatment maybe different. In FXIII deficiency due to autoimmune causes, the management usually consisted of FXIII concentrate, corticosteroids, cyclophosphamide and/or rituximab. Cases with FXIII deficiency due to consumption were treated by supplementation with FXIII concentrate only.

## References

[1] Lagrange J Wenzel P . The regulatory role of coagulation factors in vascular function. *Front Biosci (Landmark Ed)* 2019; 24:494–513. 30468669 10.2741/4731

[2] Amano S Oka K Sato Y Sano C Ohta R . Measuring factor XIII inhibitors in patients with factor XIII deficiency: a case report and systematic review of current practices in Japan. *J Clin Med* 2022; 11:1699. 35330024 10.3390/jcm11061699 PMC8955945

[3] Kleber C Sablotzki A Casu S Olivieri M Thoms KM Horter J . The impact of acquired coagulation factor XIII deficiency in traumatic bleeding and wound healing. *Crit Care* 2022; 26:69. 35331308 10.1186/s13054-022-03940-2 PMC8943792

[4] Duque P Chasco-Ganuza M Ortuzar A Almaraz C Terradillos E Perez-Rus G . Acquired FXIII deficiency is associated with high morbidity. *Thromb Haemost* 2021; 122:48–56. 33851388 10.1055/a-1481-2733

[5] Hayashi T Kadohira Y Morishita E Asakura H Souri M Ichinose A . A case of acquired FXIII deficiency with severe bleeding symptoms. *Haemophilia* 2012; 18:618–620. 22356719 10.1111/j.1365-2516.2012.02763.x

[6] Yan MTS Rydz N Goodyear D Sholzberg M . Acquired factor XIII deficiency: a review. *Transfus Apher Sci* 2018; 57:724–730. 30446212 10.1016/j.transci.2018.10.013

[7] Gagnier JJ Kienle G Altman DG Moher D Sox H Riley D . The CARE guidelines: consensus-based clinical case reporting guideline development. *Glob Adv Health Med* 2013; 2:38–43. 10.7453/gahmj.2013.008 PMC3833570 24416692

[8] Tone KJ James TE Fergusson DA Tinmouth A Tay J Avey MT . Acquired factor XIII inhibitor in hospitalized and perioperative patients: a systematic review of case reports and case series. *Transfus Med Rev* 2016; 30:123–131. 27167905 10.1016/j.tmrv.2016.04.001

[9] Katrak P Bialocerkowski AE Massy-Westropp N Kumar VS Grimmer KA . A systematic review of the content of critical appraisal tools. *BMC Med Res Methodol* 2004; 4:22. 15369598 10.1186/1471-2288-4-22 PMC521688

[10] Kun M Szuber N Katona É Pénzes K Bonnefoy A Bécsi B . Severe bleeding diatheses in an elderly patient with combined type autoantibody against factor XIII A subunit; novel approach to the diagnosis and classification of antifactor XIII antibodies. *Haemophilia* 2017; 23:590–597. 28345289 10.1111/hae.13205

[11] Bovet J Hurják B De Maistre E Katona É Pénzes K Muszbek L . Autoimmune factor XIII deficiency with unusual laboratory and clinical phenotype. *J Thromb Haemost* 2020; 18:1330–1334. 32311817 10.1111/jth.14811

[12] Wakabayashi N Nishioka H Yuzuriha S . Recurrent bleeding after head trauma caused by acquired factor XIII deficiency. *Plast Reconstr Surg Glob Open* 2022; 10:e4109. 35186643 10.1097/GOX.0000000000004109 PMC8846273

[13] Yamada Y Abe T Ochiai H Ashizuka S . Refractory duodenal bleeding ulcers successfully treated with factor XIII transfusion. *Intern Med* 2021; 60:2217–2221. 33583894 10.2169/internalmedicine.6463-20 PMC8355396

[14] Yamanishi S Kimura H Hayashi H Yamaguchi Y Fujita Y Nakai T . Acute occlusion of the ventriculoperitoneal shunt due to factor XIII deficiency-related postoperative hemorrhage: a case report. *NMC Case Rep J* 2021; 8:573–577. 35079519 10.2176/nmccrj.cr.2020-0330 PMC8769428

[15] Takashima Y Hashimoto S Kamenaga T Tsubosaka M Kuroda Y Takeuchi K . Recurrent hematomas following a revision total hip arthroplasty in acquired coagulation factor XIII deficiency. *Case Rep Orthop* 2019; 2019:4038963. 31396426 10.1155/2019/4038963 PMC6668532

[16] Jankovic M Choucair ML Hallak B Hernandez E Russo M Llor J . Massive recurrent posttonsillectomy bleedings revealing a transient factor XIII deficiency in a 10-year-old boy. A case report. *Int J Pediatr Adolesc Med* 2019; 6:55–57. 31388547 10.1016/j.ijpam.2019.05.006 PMC6676365

[17] Tone K Lalu M Kilty SJ Rosenberg E Tinmouth A . Airway compromise and perioperative management of a patient with acquired factor XIII inhibitor. *A A Case Rep* 2015; 4:120–124. 25909777 10.1213/XAA.0000000000000130

[18] Kojima M Ichinose A Souri M Osaki T Kawai H Amaki J . Successful bypass surgery for esophageal carcinoma under adequate factor XIII/13 replacement therapy in a case of intractable autoimmune hemorrhaphilia due to anti-Factor XIII/13 antibodies. *Int J Hematol* 2016; 103:341–347. 26619833 10.1007/s12185-015-1917-7

[19] Matsuoka M Majima T Onodera T Ieko M Souri M Ichinose A . Hemorrhagic-acquired factor XIII deficiency associated with tocilizumab for treatment of rheumatoid arthritis. *Int J Hematol* 2012; 96:781–785. 23070535 10.1007/s12185-012-1191-x

[20] Shimoyama S Kanisawa Y Ono K Souri M Ichinose A . First and fatal case of autoimmune acquired factor XIII/13 deficiency after COVID-19/SARS-CoV-2 vaccination. *Am J Hematol* 2022; 97:243–245. 34856014 10.1002/ajh.26426 PMC9011653

[21] d’Angelo DM Franchini S Mohn A Breda L . Factor XIII as a potential predictor of severe gastrointestinal involvement in Henoch Schoenlein purpura: a case study research. *J Paediatr Child Health* 2020; 56:1821–1823. 32297405 10.1111/jpc.14886

[22] Di Micco P Gussoni G Pieralli F Campanini M Dentali F Fontanella A . Acquired factor XIII deficiency inducing recurrent and fatal bleeding, description of a case. *J Blood Med* 2020; 11:43–45. 32099500 10.2147/JBM.S232115 PMC6996541

[23] Murata M Inatomi O Ono K Imai T Iwasa M Kawahara M . A case of life-threatening small intestinal bleeding accompanied by lower coagulation factor XIII activity. *Clin J Gastroenterol* 2020; 13:1178–1182. 32710383 10.1007/s12328-020-01195-4

[24] Soto I Bernardo A Arias T Ramón C Noval I Palomo C . The first case report of a patient with acquired factor XIII deficiency in the context of autoimmune encephalitis. *Haemophilia* 2017; 23:e461–e464. 28664679 10.1111/hae.13281

[25] Rabik CA Atkinson MA Sule S Strouse JJ . Treatment of an acquired Factor XIII inhibitor in an adolescent with systemic lupus erythematosus and renal failure. *Transfusion* 2017; 57:2159–2163. 28707410 10.1111/trf.14185

[26] Pénzes K Rázsó K Katona É Kerényi A Kun M Muszbek L . Neutralizing autoantibody against factor XIII A subunit resulted in severe bleeding diathesis with a fatal outcome – characterization of the antibody. *J Thromb Haemost* 2016; 14:1517–1520. 27208811 10.1111/jth.13367

[27] Nixon CP Prsic EH Guertin CA Stevenson RL Sweeney JD . Acquired factor XIII inhibitor associated with mantle cell lymphoma. *Transfusion* 2017; 57:694–699. 27917497 10.1111/trf.13947

[28] Kotake T Souri M Takada K Kosugi S Nakata S Ichinose A . Report of a patient with chronic intractable autoimmune hemorrhaphilia due to antifactor XIII/13 antibodies who died of hemorrhage after sustained clinical remission for 3 years. *Int J Hematol* 2015; 101:598–602. 25663511 10.1007/s12185-015-1754-8

[29] Boehlen F Casini A Chizzolini C Mansouri B Kohler HP Schroeder V . Acquired factor XIII deficiency: a therapeutic challenge. *Thromb Haemost* 2013; 109:479–487. 23306660 10.1160/TH12-08-0604

[30] Mitchell JL Wright S Kazi S Watson HG Mutch NJ . Defective 2 antiplasmin cross-linking and thrombus stability in a case of acquired factor XIII deficiency. *Br J Haematol* 2017; 178:794–799. 28516512 10.1111/bjh.14759

[31] Takamatsu N Manabe H Wada K Hirano T Chikawa T Sairyo K . Successful treatment of intractable pseudomeningocele with FXIII deficiency by surgery and FXIII replacement therapy: a case report. *Int J Surg Case Rep* 2022; 92:106851. 35278986 10.1016/j.ijscr.2022.106851 PMC8917295

[32] Uchida E Watanabe K Arai R Yamamoto M Souri M Osaki T . Autoimmune hemorrhaphilia resulting from autoantibody against the a subunit of factor XIII. *Intern Med* 2015; 54:2383–2387. 26370866 10.2169/internalmedicine.54.4791

[33] Lichter Y Badelbayov T Shalev I Schvartz R Szekely Y Benisty D . Low FXIII activity levels in intensive care unit hospitalized COVID-19 patients. *Thromb J* 2021; 19:79. 34736472 10.1186/s12959-021-00333-3 PMC8567130

[34] Kessel R Hu C Shore-Lesserson L Rand J Manwani D . A child with acquired factor XIII deficiency: case report and literature review. *Haemophilia* 2013; 19:814–826. 23607876 10.1111/hae.12145

[35] Mokuda S Murata Y Sawada N Matoba K Yamada A Onishi M . Tocilizumab induced acquired factor XIII deficiency in patients with rheumatoid arthritis. *PLoS One* 2013; 8:e69944. 23936360 10.1371/journal.pone.0069944 PMC3731329

[36] Beckman JD Kasthuri RS Wolberg AS Ma AD . Challenges in diagnosis and management of acquired factor XIII (FXIII) inhibitors. *Haemophilia* 2018; 24:e417–e420. 30144219 10.1111/hae.13603 PMC6390178

[37] Feugray G Buchbinder N Buchonnet G Fenneteau O Billoir P Le Cam Duchez V . Acquired factor XIII deficiency in a child with pure erythroid leukemia. *Pediatr Blood Cancer* 2021; 68:e28890. 33484080 10.1002/pbc.28890

[38] Tsuda M Kiyasu J Sugio K Hidaka D Ikeda M Fujioka E . Spontaneous splenic rupture accompanied by hepatic arterial dissection in a patient with autoimmune haemorrhaphilia due to antifactor XIII antibodies. *Haemophilia* 2016; 22:e314–e317. 27167212 10.1111/hae.12940

[39] Gonçalves E Lopes da Silva R Varandas J Diniz MJ . Acute promyelocytic leukaemia associated Factor XIII deficiency presenting as retro-bulbar haematoma. *Thromb Res* 2012; 129:810–811. 22483775 10.1016/j.thromres.2012.03.008

[40] Ferretti A Baldacci E Fazio F Abbruzzese R Barone F De Luca ML . Acquired FXIII deficiency and AL amyloidosis: a case of a rare association. *Transfus Apher Sci* 2020; 59:102903. 32839100 10.1016/j.transci.2020.102903

[41] Tang N Li D Wang X Yang J . Concurrent hematoma and venous thrombosis in a patient with autoimmune acquired factor XIII deficiency. *Int J Lab Hematol* 2020; 42:e4–e6. 31132215 10.1111/ijlh.13053

[42] Ngo Sack F Galinat H Egreteau PY Mollard LM Fortin H Berthou C . Efficacy of rituximab in acquired factor XIII inhibitor after arterial rFVIIa-induced thrombosis. *Haemophilia* 2013; 19:e93–e94. 23205641 10.1111/hae.12069

[43] Tha MH Tien SL . Acquired factor XIII deficiency: still a clinical challenge in the era of novel therapy. *Haemophilia* 2014; 20:e104–e105. 24354481 10.1111/hae.12314

[44] Levy JH Greenberg C . Biology of Factor XIII and clinical manifestations of Factor XIII deficiency. *Transfusion* 2013; 53:1120–1131. 22928875 10.1111/j.1537-2995.2012.03865.x

[45] Hetz M Juratli T Tiebel O Giesecke MT Tsitsilonis S Held HC . Acquired factor XIII deficiency in patients with multiple trauma. *Injury* 2023; 54:1257–1264. 36577625 10.1016/j.injury.2022.12.021

[46] Ichinose A Kohler HP Philippou H . Factor XIII and Fibrinogen SSC Subcommittee of the ISTH Recommendation for ISTH/SSC Criterion 2015 for autoimmune acquired factor XIII/13 deficiency. *Thromb Haemost* 2016; 116:772–774. 27439329 10.1160/TH16-05-0362

[47] Ichinose A Osaki T Souri M . Pathological coagulation parameters in as many as 54 patients with autoimmune acquired factor XIII deficiency due to antifactor XIII autoantibodies. *Haemophilia* 2021; 27:454–462. 33847063 10.1111/hae.14298

[48] Osaki T Souri M Ichinose A . Important roles of the human leukocyte antigen class I and II molecules and their associated genes in the autoimmune coagulation factor XIII deficiency via whole-exome sequencing analysis. *PLoS One* 2021; 16:e0257322. 34506591 10.1371/journal.pone.0257322 PMC8432773

[49] Muszbek L Katona É . Diagnosis and management of congenital and acquired FXIII deficiencies. *Semin Thromb Hemost* 2016; 42:429–439. 27071048 10.1055/s-0036-1572326

